# Epstein-Barr Virus Immortalization of Human B-Cells Leads to Stabilization of Hypoxia-Induced Factor 1 Alpha, Congruent with the Warburg Effect

**DOI:** 10.1371/journal.pone.0042072

**Published:** 2012-07-27

**Authors:** Suhas Darekar, Konstantinos Georgiou, Mariya Yurchenko, Surya Pavan Yenamandra, Georgia Chachami, George Simos, George Klein, Elena Kashuba

**Affiliations:** 1 Department of Microbiology, Tumor and Cell Biology (MTC), Karolinska Institutet, Stockholm, Sweden; 2 R.E. Kavetsky Institute of Experimental Pathology, Oncology and Radiobiology, NASU, Kyiv, Ukraine; 3 School of Medicine, University of Thessaly, Larissa, Greece; 4 Institute of Biomedical Research and Technology (BIOMED), Larissa, Greece; The University of Hong Kong, China

## Abstract

**Background:**

Epstein-Barr virus (EBV) encodes six nuclear transformation-associated proteins that induce extensive changes in cellular gene expression and signaling and induce B-cell transformation. The role of HIF1A in EBV-induced B-cell immortalization has not been previously studied.

**Methods and Findings:**

Using Western blotting and Q-PCR, we found that HIF1A protein is stabilized in EBV-transformed lymphoblastoid cells. Western blotting, GST pulldown assays, and immunoprecipitation showed that EBV-encoded nuclear antigens EBNA-5 and EBNA-3 bind to prolylhydroxylases 1 and 2, respectively, thus inhibiting HIF1A hydroxylation and degradation. Immunostaining and Q-PCR showed that the stabilized HIF1A translocates to the nucleus, forms a heterodimer with ARNT, and transactivates several genes involved in aerobic glycolysis. Using biochemical assays and Q-PCR, we also found that lymphoblastoid cells produce high levels of lactate, lactate dehydrogenase and pyruvate.

**Conclusions:**

Our data suggest that activation of the aerobic glycolytic pathway, corresponding to the Warburg effect, occurs in EBV-transformed lymphoblastoid cells, in contrast to mitogen-activated B-cells.

## Introduction

Epstein-Barr virus (EBV) is a ubiquitous human gamma herpesvirus that causes lymphoproliferative disease in immunosuppressed patients and is associated with Burkitt's lymphoma, Hodgkin's lymphoma, other B- and T-cell lymphomas, nasopharyngeal carcinoma (NPC) and some gastric carcinomas [1]. EBV can infect B-cells *in vitro* and convert them into immortalized lymphoblastoid cell lines (LCLs). LCLs express six nuclear proteins (EBNA-1–6) and three latent membrane proteins (LMP-1, -2A and -2B). This growth transformation program is referred to as Latency III. Six of the proteins, EBNA-1, -2, -3, -5, -6 and LMP-1, are required for efficient B-cell transformation [1].

Expression of the six transformation-associated proteins leads to extensive changes in cellular gene expression and signaling. The EBNAs interact with a variety of cellular proteins and play different roles in the viral strategy. EBNA-1 is essential for the maintenance of viral episomes and regulates viral promoter usage; EBNA-2 activates the viral LMP-1 promoter and a number of cellular genes, such as *CD23, C-MYC, cyclin D1* and *HES*. EBNA-5 enhances EBNA-2-dependent transcription and inactivates p53 by forming a trimolecular complex with MDM2 and p53 [2]. EBNA-6 is involved in chromatin remodeling and the regulation of the RB pathway (for a review, see [3]).

The EBNA-3 family proteins (EBNA-3, -4 and -6) regulate the transcription of viral and cellular genes and are involved in the control of G_2_/M transition (reviewed in [3]). EBNA-3 also participates in the regulation of transactivation by several nuclear receptors. It binds to aryl hydrocarbon receptor (AHR) (NP_001612) and its regulatory protein XAP-2 (NP_003968) [4] and thereby enhances the constitutive and ligand- (e.g., TCDD) activated transcription of AHR-responsive genes [5]. Moreover, EBNA-3 blocks the transcription of VDR-responsive genes, and thereby protects LCLs against the growth arrest and/or apoptosis induced by the interaction of vitamin D3 with its receptor (VDR, NP_000367) [6].

Virally encoded membrane proteins may also regulate the activity of nuclear receptors. LMP1 can enhance the synthesis of HIF1A (NP_001521) and the expression of HIF1A-responsive genes in an NPC-derived cell line [7], which could be attributed to the enhanced degradation of prolylhydroxylases 1 and 3, mediated by SIAH1 [8].

HIF1A is frequently overexpressed in solid tumors where its levels correlate with tumor progression and poor prognosis in non-small cell lung and colorectal carcinomas, fibrosarcoma and tumors of brain, breast, esophagus, stomach, prostate, ovary, uterus and cervix (reviewed in [9]). HIF1A is one of the subunits of the HIF1 transcription factor. When heterodimerized with aryl hydrocarbon receptor nuclear translocator (ARNT or HIF1B, NP_001659), it activates several genes that mediate oxygen delivery and oxygen utilization under hypoxic conditions. Normally, HIF1A is hydroxylated by prolylhydroxylases 1 and 2 (PHD1, NP_542770; PHD2, NP_071334). The hydroxylated form is recognized by the Von Hippel–Lindau (VHL, NP_000542) protein, an E3 ubiquitin ligase, and degraded by the proteasome machinery.

The expression level of HIF1A in lymphoblastoid cells and its function in EBV-induced B-cell transformation have not been previously studied. Given that the malignant or premalignant behavior of EBV-activated B-cells has been a matter of some controversy among pathologists, it is of considerable interest to establish whether the virally activated immunoblasts show a Warburg effect, as with most malignant cells and in contrast to mitogen-activated B-blasts.

We here report that HIF1A is stabilized in EBV-transformed lymphoblastoid cells at the protein level. Two virally encoded and growth transformation-associated proteins, EBNA-5 and EBNA-3, bind to prolylhydroxylases 1 and 2, respectively, and interfere with their enzymatic activity. HIF1A is known to transactivate several genes involved in aerobic glycolysis (for a review, see [10]). We also found that lymphoblastoid cells produce high levels of lactate, lactate dehydrogenase and pyruvate. This suggests the activation of the glycolytic pathway, corresponding to the Warburg effect. No such activities were found in mitogen-activated B-cells.

## Methods

### Plasmids

Cloning of GST2TK-EBNA-5, GST-EBNA-5(2W), RedDS-EBNA-5, GST-EBNA-3, GST-CTEBNA-3 and FLAG-EBNA-3 was described previously. The EBNA-5 constructs contained four W_1_W_2_ repeats and the unique C-terminal domain (Y) except GST-EBNA-5(2W), which contained only two W_1_W_2_ repeats. GFP-HIF1A, GST-HIF1A and GST-ARNT were constructed as described previously [11], [12]. All the constructs were verified by sequencing using a capillary Applied Biosystems sequencing machine (PerkinElmer, Wellesley, MA).

### Cell lines and transfections

The MCF7 breast carcinoma cell line and LCLs (described in [2], [6]) were cultured at 37°C in Iscove's medium containing 10% fetal bovine serum and appropriate antibiotics. Periodic staining with Hoechst 33258 (Sigma-Aldrich, St. Louis, MO) confirmed the absence of mycoplasma. MCF7 cells were grown on coverslips and transfected using Lipofectamine Plus Reagent (Life Technologies, Carlsbad, CA), according to the manufacturer's protocol. Peripheral B-cells were isolated from buffy coat (Karolinska Hospital, Stockholm) on Lymphoprep gradients. Two subsequent rounds of E-rosetting removed the T-cells. The B95.8 strain of EBV was used for B-cell infection to establish LCLs. For virus-unrelated B-cell activation, the cells were exposed to anti-CD40 antibody (Nordic Biosite AB, Täby, Sweden; 1 µg/ml) and interleukin 4 (IL4) (ImmunoTools GmbH, Friesoythe, Germany; 25 ng/ml). All cells were activated by the mitogen, while approximately 40% of cells were infected after 24 h, as shown by immunostaining with anti-EBNA-2. Cells were deposited on glass slides using a Cytospin cytocentrifuge.

### GST protein production and GST pulldown assay

GST fusion proteins were isolated from bacterial cultures (BL21 DE3 strain, Invitrogen, Carlsbad, CA), according to the manufacturer's protocol. These proteins were treated with DNAse and RNAse A to reduce nonspecific interference and purified on GST Sephacryl beads (GE Healthcare Bio-Sciences AB, Uppsala, Sweden). A GST pulldown assay was performed as described previously [13].

### Western blotting

Total cell lysates were prepared using the NP40 lysis buffer (1% NP40, 150 mM NaCl, 50 mM Tris, pH = 8) with a protease inhibitor cocktail (Roche AB, Stockholm, Sweden) and containing BSA (0.5%(w/v)) as a nonspecific competitor. After SDS–PAGE, proteins were transferred to nitrocellulose and probed with rabbit antibodies against HIF1A, HIF1A-OH (Cell Signaling Technology, Inc., Billerica, MA), PHD1, PHD2 (Thermo Scientific, Rockford, IL) and EBNA-3 (AsLa Ltd, Riga, Latvia) and with mouse antibodies against HIF1A (Life Technologies), EBNA-5 (DAKO, Glostrup, Denmark) and actin (Sigma-Aldrich). Secondary antibodies (anti-rabbit and anti-mouse IgG HRP conjugated) were from GE Healthcare Bio-Sciences AB.

### Immunoprecipitations

Antibodies (5 µg of rabbit anti-EBNA-3, mouse anti-EBNA-5 and normal mouse serum (DAKO)) were coupled to CNBr-Sepharose 4 Fast Flow, according to the manufacturer's protocol (GE Healthcare Bio-Sciences AB). These supports were used with whole cell lysates typically prepared from 10^7^ lymphoblastoid cells. After extensive washing with NP40 lysis buffer and PBS, immune complexes were eluted by heating (94°C) and analyzed on SDS–PAGE.

### Immunostaining and imaging

Immunostaining and digital image capture were performed as described elsewhere. Briefly, cells on coverslips were fixed in a 1∶1 mixture of cold methanol and acetone at −20°C. After rehydration in PBS, cells were stained with rabbit anti-PHD1, anti-PHD2 and mouse anti-FLAG (Rockland, Gilbertsville, PA) antibodies. The following secondary antibodies were used: goat anti-rabbit biotinylated, AMKA streptavidin and horse anti-mouse Texas red conjugated (BD Biosciences Pharmingen, San Jose, CA). Images were captured using a DAS microscope, Leitz DM RB, with a dual-mode CCD camera, C4880 (Hamamatsu, Japan).

### Real-time PCR

Total RNA was purified from cells (with and without treatment with NiCl_2_ (1 mM) for 20 h) using the RNA Purification Kit (Fermentas, Hanover, MD). One microgram of total RNA was used for the cDNA synthesis using a First Strand cDNA Synthesis Kit (Fermentas); for real-time PCR, the total reaction volume was 20 µl and the primer concentration was adjusted to a final concentration of 3 µM. Real-time PCR was performed using SYBR Green Master Mix on a 7900 machine (Applied Biosystems, Foster City, CA). Primers for HIF1A1-responsive genes were the following: *GLUTI* (NM_006516) F-AAGGTGATCGAGGAGTTCTACA, R-ATGCCCCCAACAGAAAAGATG; *hexokinase-2* (*HK-2*) (NM_000189), F-GAGCCACCACTCACCCTACT, R-ACCCAAAGCACACGGAAGTT; PFKL (X16930) F-CTGGGA GAACTTCATGTGTGAG, R-TAGCTGGACGAGATGGGCT; *LDHA1* (NM_005566) F-CTCCAAGCTGGTCATTATCACG, R-AGTTCGGGCTGTATTTTACAACA; *PDK1* (NM_002610) F-TCCTGGACTTCGGATCAGTGA, R-CGGATGGTGTCCTGAGA AGATT; *PGK1* (NM_000291) F-TTAAAGGGAAGCGGGTCGTTA, R-TCCATTGTCC AAGCAGAATTTGA; *MCT4* (NM_004207) F-TGTGTGCGTGAACCGCTTT, R-AAAC CCAACCCCGTGATGAC; *PKLR* (D13243) F-CTTCGGTCATGGGTCTCTAAGT; *GAPDH* (NM_002046) F-ATGGGGAAGGTGAAGGTCG, R-GGGGTCATTGATGGC AACAATA. Primers for the *HIF1A* gene were F-ATCCATGTGACCATGAGGAAATG and R-CTCGGCTAGTTAGGGTACACTT.

As an internal control for standardization, a gene encoding TATA-binding protein (*TBP*, NM_003194) was used with the following primers: F-TTTCTTGCCAGTCTGG AC, R-CACGAACCACGGCACTGATT.

The PCR cycling conditions were the following: 10 min at 95°C, 40 cycles of 10 s at 95°C and 1 min at 60°C. Applied Biosystems 7900 systems software was used for analysis. Ct values were determined for the internal control (TBP) and for the test genes at the same threshold level in the exponential phase of the PCR curves. Relative quantification (comparative Ct (ΔΔCt) method) was used to compare the expression level of the test genes with the internal control. Dissociation curve analysis was performed after every run to check the specificity of the reaction. Three or four reactions (each in triplicate) were run for each gene and the standard deviation was calculated.

### Biochemical assays

Activated B-cells, EBV-infected B-cells and LCLs were assayed for l-lactate, pyruvate and lactate dehydrogenase. Peripheral blood B-cells (5×10^6^) were activated with anti-CD40 and IL4, or infected with EBV and grown for 5 days. LCLs (5×10^6^) were also grown for 5 days. Colorimetric assays were as described by Bioassay Systems (Hayward, CA) for medium lactate (l-Lactate Assay Kit, ECLC-100), pyruvate (Pyruvate Assay Kit, EPYR-100) and lactate dehydrogenase (Lactate Dehydrogenase Kit, DLDH-100). To measure lactate concentration, the cell culture medium was collected. For pyruvate and lactate dehydrogenase assays, 3×10^6^ cells were sonicated in 300 µl of 100 mM potassium phosphate/2 mM EDTA (pH 7.0) and the absorbance of standards and unknowns was measured on a microplate reader at 565 nm.

## Results

### HIF1A protein is stabilized in LCLs

**Figure 1:**
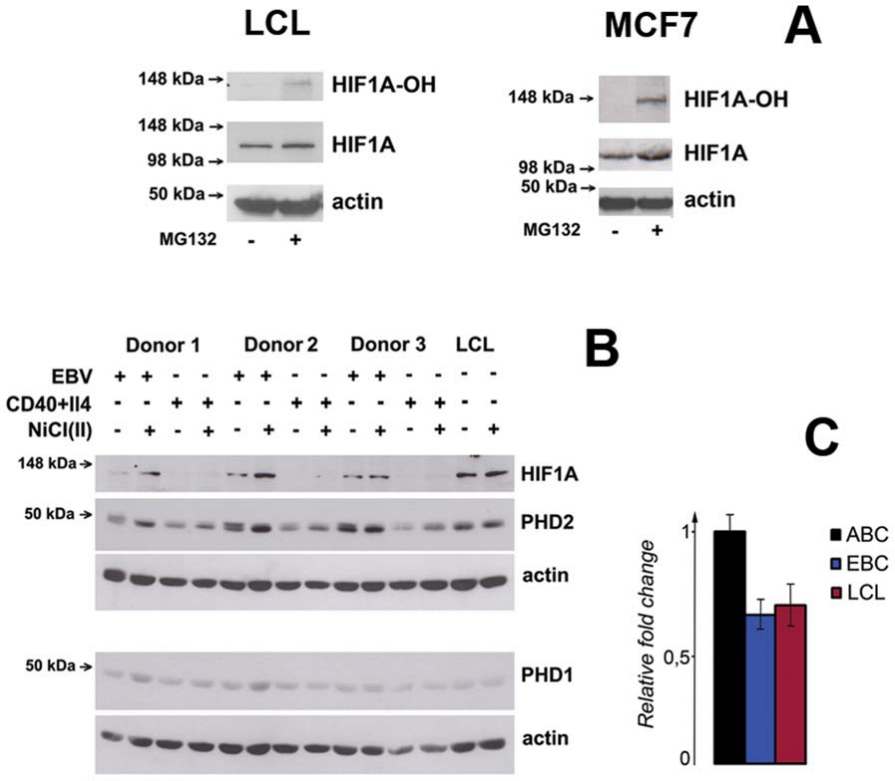
Expression level of HIF1A in EBV-infected and mitogen-activated B-cells, and in LCLs. **A –** Western blotting of HIF1A and its hydroxylated form in LCLs (left panel). The membrane was probed with mouse antibody against HIF1A and the rabbit serum against the hydroxylated form of HIF1A (HIF1A-OH). Notice the absence of HIF1A-OH in LCLs (first lane) and the lack of effect of proteasome inhibition. In contrast, high levels of hydroxylated HIF1A were detected in control MCF7 cells upon proteasome inhibition (right panel). **B –** Western blotting of whole cell lysates of CD40+IL4-activated B-cells, freshly EBV-infected B-cells and LCLs. Cells were treated with 1 mM NiCl_2_, mimicking hypoxia-like conditions. Notice that HIF1A, PHD1 and PHD2 levels in LCLs did not change upon treatment with NiCl_2_, in contrast to freshly infected cells. **C –** *HIF1A* expression in CD40+IL4-activated (ABC), freshly EBV-infected (EBC) B-cells and LCLs (LCL) measured by Q-PCR.

HIF1A levels in lymphoblastoid cells were determined by Western blotting of whole cell lysates for HIF1A and its hydroxylated form (HIF1A-OH). Because HIF1A is usually hydroxylated and degraded very fast under normal conditions, HIF1A was also assayed after blocking proteasomal degradation with MG132. A strong HIF1A signal was obtained with LCL lysates, prepared with or without MG132 (Figure 1A, left panel). This suggests that HIF1A protein is stabilized in LCLs. To answer the question whether EBV plays a role in HIF1A stabilization, HIF1A levels were measured in freshly infected B-cells and in CD40+IL4-activated B-cells by Western blotting. Cells were collected after 5 days of activation/infection, and the levels of HIF1A and prolylhydroxylases 1 and 2 were compared in NiCl_2_-treated and -untreated cells (Figure 1B). Ni^+2^ and Co^2+^ ions can replace iron cations in the active catalytic center of HIF1A prolylhydroxylases, and thereby block their activity [14]. This leads to HIF1A protein stabilization, because only the hydroxylated form is recognized by VHL, the E3 ubiquitin ligase for HIF1A [15].

Importantly, the levels of HIF1A and prolylhydroxylases 1 and 2 in LCLs did not change under conditions mimicking hypoxia, indicating that HIF1A is stabilized in LCLs. Q-PCR was performed to check whether the high HIF1A levels were simply due to enhanced transcription. This was not the case because *HIF1A* mRNA expression was similar in primary peripheral blood B-cells, mitogen-activated B-cells and B-cells freshly infected with EBV (Figure 1C).

Together with the absence of hydroxylated HIF1A, our findings indicate that the HIF1A protein is stabilized in lymphoblastoid cells.

#### EBNA-3 forms a complex with HIF1A

**Figure 2:**
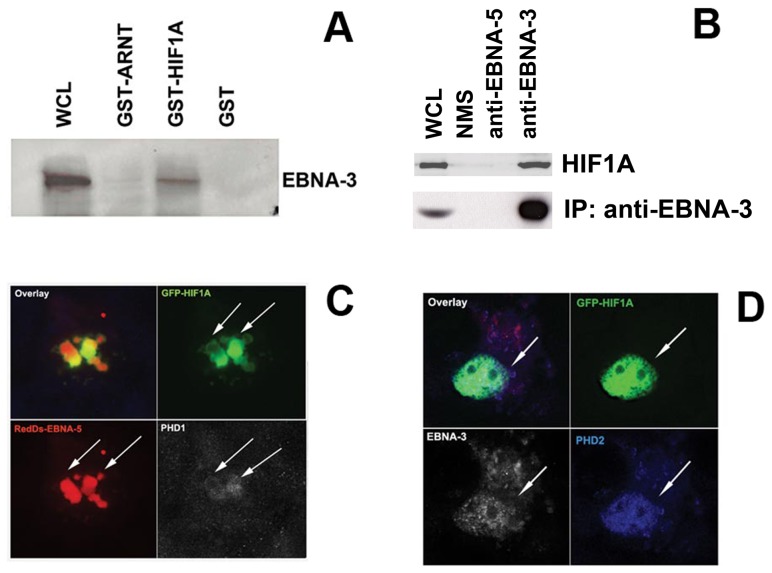
HIF1A binds to EBNA-3, and PHDs 1 and 2 colocalize with EBNA-5 and EBNA-3, respectively. **A –** GST pulldown assay and Western blotting from lymphoblastoid cell lysates. EBNA-3 was detected in a complex with HIF1A protein. As a positive control, 5% of the crude lysate was loaded. **B –** Immunoprecipitation and Western blotting from lymphoblastoid cell lysates. HIF1A was detected in a complex with EBNA-3, but not with EBNA-5, on the surface of CNBr beads with immobilized anti-EBNA-3 and anti-EBNA-5 antibodies, respectively. As a positive control, 7% of the crude lysate was loaded. **C –** Immunostaining of MCF7 cells transfected with GFP-HIF1A (green) and RedDs-EBNA-5 (red). Rabbit anti-PHD1 antibody, followed by biotinylated goat anti-rabbit antibody and AMKA streptavidin (white). EBNA-5 and HIF1A partially colocalize in the nucleus. In doubly transfected cells, the normally cytoplasmic PHD1 is located in the nucleus, colocalized with GFP-HIF1A and RedDS-EBNA-5. **D –** MCF7 cells transfected with GFP-HIF1A (green) and FLAG-EBNA-3 (white) were stained with rabbit anti-PHD1 antibody, followed by biotinylated goat anti-rabbit antibody and AMKA streptavidin (blue). The mainly cytoplasmic PHD2 redistributed to the nucleus in doubly transfected cells, colocalizing with GFP-HIF1A and FLAG-EBNA-3.

To test whether the EBV-encoded transforming proteins, EBNA-3, -5, and -6, are involved in stabilization of HIF1A, a GST pulldown assay was performed, using glutathione *S*-transferase (GST) alone and GST fusion proteins with HIF1A or ARNT immobilized on the surface. Proteins from whole lymphoblastoid cell lysates were captured by the support and then analyzed by Western blotting. The membranes were probed with mouse anti-EBNA-3, anti-EBNA-6 and anti-EBNA-5. This showed that EBNA-3 was bound specifically by GST-HIF1A, whereas EBNA-5 and EBNA-6 did not bind to GST-HIF1A or GST-ARNT (Figure 2A).

Immunoprecipitates were prepared from LCL lysates using either anti-EBNA-3 or anti-EBNA-5 coupled to CNBr Sepharose beads and normal mouse serum (NMS) as a control for the lysates. Western blotting analysis showed coprecipitation of HIF1A and EBNA-3 but not with EBNA-5 in this preparation (Figure 2B).

#### HIF1A colocalizes with EBNA-3 and EBNA-5 in the nucleus

To monitor the cellular distribution of HIF1A in the presence of EBNA-3 and EBNA-5, cells of the MCF7 breast carcinoma line were transfected with plasmids encoding GFP-HIF1A, RedDs-EBNA-5 and FLAG-tagged EBNA-3. After fixation and rehydration with PBS, GFP-HIF1A+RedDs-EBNA-5-transfected cells were stained with rabbit sera against PHD1 and PHD2, followed by goat anti-rabbit biotinylated antibody and AMKA streptavidin. Cells expressing GFP-HIF1A and RedDs-EBNA-5 showed a high degree of nuclear colocalization of these proteins (Figure 2C). In the doubly transfected cells, PHD1 (white), which is normally found in the cytoplasm, was located in the nucleus colocalized with both HIF1A and EBNA-5. This suggests that EBNA-5 may form a complex with PHD1 and so impair its enzymatic activity. Immunostaining showed much more limited colocalization of PHD2 with HIF1A and EBNA-5 in the nucleus.

MCF7 cells were also cotransfected with GFP-HIF1A and FLAG-EBNA-3 plasmids and stained for PHD1 and PHD2 (in blue, Figure 2D). Subsequently, the cells were stained with mouse monoclonal anti-FLAG and horse anti-mouse Texas Red conjugated antibodies (red signal) to visualize FLAG-tagged EBNA-3. The staining pattern for PHD1 and PHD2 was similar to that described above in MCF7 cells transfected with EBNA-5 and HIF1A. The normally cytoplasmic PHD2 (blue signal) was found in the nucleus, where it colocalized with GFP-HIF1A (green signal) and EBNA-3 (in white; Figure 2D). PHD1 was also colocalized with GFP-HIF1A in the nucleus, but to a lesser extent than PHD2.

#### Prolylhydroxylase 1 and 2 bind to EBNA-5 and EBNA-3, respectively

**Figure 3:**
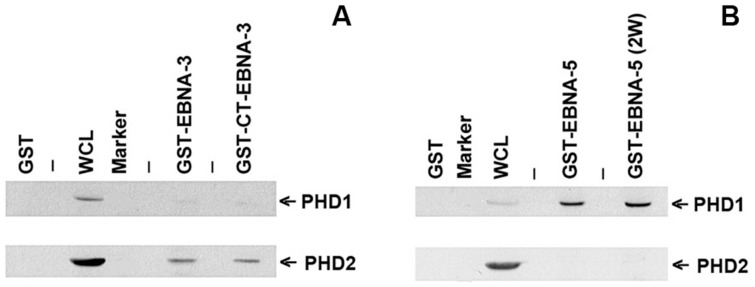
PHDs 1 and 2 bind to EBNA-5 and EBNA-3, respectively. **A –** Western blotting following the GST pulldown assay from lymphoblastoid cell lysates. PHD2 and a small portion of PHD1 were precipitated on the beads with GST-EBNA-3 from cell lysate. Positive control shows 15% the crude lysate. **B –** Western blotting following the GST pulldown assay from LCL lysates. PHD1 was precipitated on the support with immobilized GST-EBNA-5 protein from the lymphoblastoid cell lysate. Positive control shows 15% of the crude lysate.

To verify that EBNA-3 may form a complex with PHD2 and EBNA-5 with PHD1, a GST pulldown assay was performed using lymphoblastoid cell lysates. GST tagged with intact EBNA-3, the C-terminal region of EBNA-3, EBNA-5 containing either 2 or 4 W_1_W_2_ repeats, and GST alone on the beads were used in a pulldown assay. Substantial amounts of PHD2 were precipitated with EBNA-3, whereas PHD1 formed complexes with EBNA-5 (Figure 3A and B).

These results indicate that HIF1A is stabilized in LCLs because of a lack of hydroxylation, which may in turn result from an inhibition of the catalytic activity of PHDs due to binding of cognate EBNAs.

#### Nuclear HIF1A pool is enriched in LCLs

**Figure 4:**
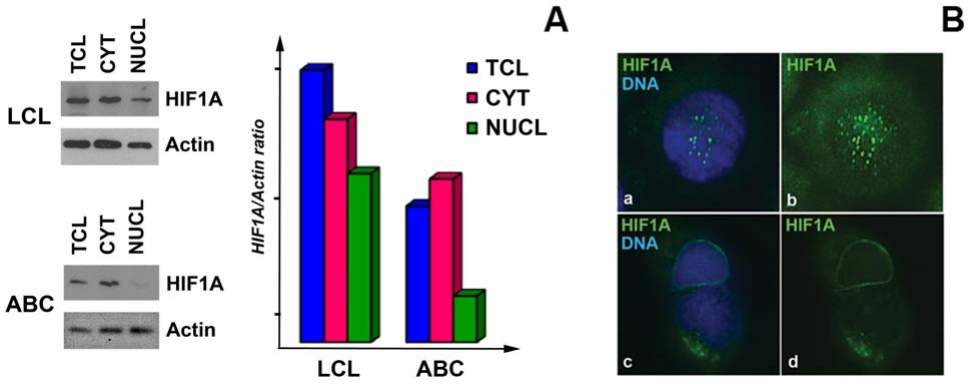
HIF1A is localized to the nucleus and cytoplasm in LCLs, in contrast to mitogen-activated B-cells. **A –** Western blotting of cellular subfractions. The membrane was probed with mouse anti-HIF1A and anti-actin antibodies (left panel). Nuclear HIF1A was detected in the lysates of LCL; however, no nuclear signal for HIF1A was detected in mitogen-activated cells. The HIF1A/actin ratio for all the probes was calculated (right panel). **B –** Immunostaining of LCLs and mitogen-activated cells with mouse anti-HIF1A antibody (green signal). Nuclear DNA is stained in blue. Notice the presence of nuclear HIF1A signal in LCL (panels **a** and **b**) and its absence in the mitogen-activated cells (**c** and **d**).

To test directly whether the level of HIF1A is increased in the nucleus of LCLs, we separated cytoplasmic and nuclear fractions from LCLs and CD40+IL4-activated cells. Western blotting with anti-HIF1A antibody showed an increase in nuclear HIF1A in LCLs (Figure 4A), and similar conclusions were drawn from immunostaining experiments (Figure 4B). Conversely, the HIF1A signal (green) was observed mainly in the cytoplasm of mitogen-activated B-cells, while in LCL it showed both a cytoplasmic and a nuclear distribution.

#### HIF1A is constitutively transcriptionally active in LCLs

**Figure 5:**
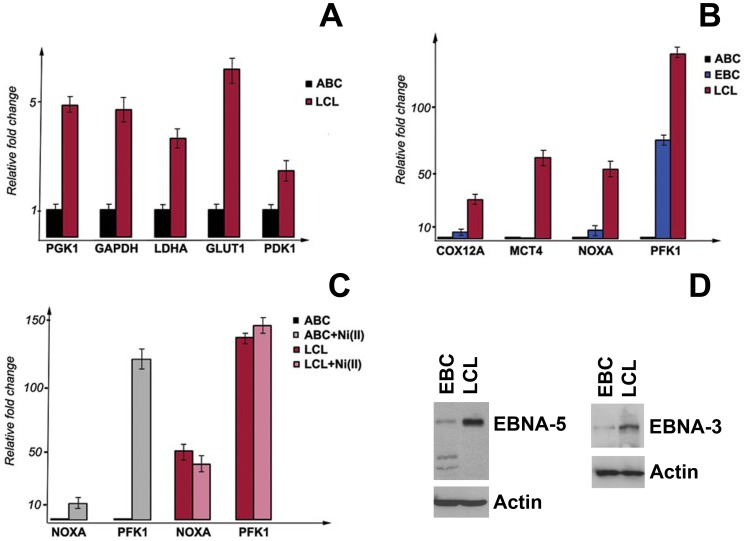
Expression of HIF1A-responsive genes in EBV-infected and mitogen-activated B-cells and LCLs. Expression of HIF1A-responsive genes, assessed by Q-PCR. CD40+IL4-activated (ABC) and freshly EBV-infected (EBC) B-cells were compared with LCLs. Untreated and cells treated with NiCl_2_ were compared. Notice that many genes are upregulated in LCL and freshly EBV-infected cells, compared with CD40+IL4-activated B-cells.

To test whether the transactivating ability of HIF1A was inhibited in the LCLs, Q-PCR was performed for HIF1A-responsive genes (Figure 5). LCLs were compared with both CD40+IL4-activated and freshly EBV-infected B-cells. Given that EBV-infected immunoblasts are not normally exposed to hypoxia either *in vitro* or in their natural niche *in vivo*, and given the involvement of HIF1A in glycolysis, we examined the expression of several genes of the glycolytic pathway (Figure 5).

*GLUT1* (glucose transporter), *LDHA* (lactate dehydrogenase), *PDK1* (pyruvate dehydrogenase kinase), *PGK1* (phosphoglycerate kinase), *PFKL* (phosphofructokinase), *SLC16A3* (*MCT4*, monocarboxylate transporter), *HK1* (hexokinase) and *PKLR* (pyruvate kinase) were expressed at levels 5–150-fold higher in freshly EBV-infected cells and in LCLs than in CD40+IL4-activated B-cells. These genes encode essential enzymes involved in glycolysis: GLUT1 controls glucose transport; PDK1 catalyzes oxidative decarboxylation of pyruvate; and LDHA controls a step in anaerobic glycolysis by catalyzing the conversion of l-lactate and NAD into pyruvate and NADH, respectively.

#### LCLs show a “Warburg effect”

**Figure 6:**
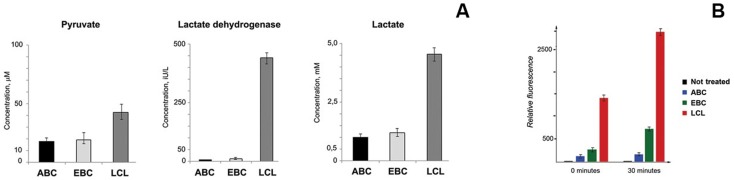
Biochemical analysis of EBV-infected and mitogen-activated B-cells, and LCLs. **A –** Concentration of pyruvate (left panel), lactate (right panel) and lactate dehydrogenase catalytic activity (middle panel) in CD40+IL4-activated (ABC), freshly EBV-infected (EBC) B-cells and LCLs. Notice increased values in LCLs compared with CD40+IL4-activated B-cells, while EBV-infected B-cells showed intermediate values. **B –** Production of reactive oxygen species in CD40+IL4-activated (ABC), freshly EBV-infected (EBC) B-cells and LCLs. Notice the increased ROS concentration in LCLs, compared with CD40+IL4. Freshly EBV-infected B-cells showed intermediate levels of ROS.

To test whether LCLs show a “Warburg effect” (i.e., a preference for aerobic glycolysis, similar to that seen in solid tumors), we measured lactate and pyruvate levels and LDHA enzymatic activity in cultured cells. CD40+IL4-activated cells were compared with freshly EBV-infected B-cells and LCLs cultured under the same conditions. The concentrations of lactate and pyruvate were 5- and 3-fold higher in the LCLs than in mitogen-activated B-cells. The catalytic activity of LDHA was at least 50-fold higher in LCLs, compared with CD40+IL4-activated B-cells. EBV-infected B-cells showed intermediate values for lactate, pyruvate and lactate dehydrogenase (Figure 6A).

Because enhanced ATP synthesis leads to elevated levels of reactive oxygen species (ROS), their levels were assayed in all three groups of cells. Higher levels of ROS were detected in LCLs than in CD40+IL4-activated B-cells, suggesting increased energy production by lymphoblastoid cells (Figure 6B). Freshly EBV-infected cells showed intermediate values.

## Discussion

HIF1A is a transcription factor characterized by a basic helix-loop-helix (PAS) domain at the N terminus [14]. It is powerfully induced by hypoxia at both mRNA and protein levels [14]. Under normal conditions, the HIF1A protein level is low because of proteasome-dependent degradation. At high oxygen levels, a few prolines (e.g., residues 402 and 564) are hydroxylated [16], [17], leading to the recognition of HIF1A by its E3 ligase, VHL [18], followed by proteasomal degradation.

Our findings show that HIF1A is stabilized in EBV-transformed B-cells and, given that mRNA levels are not changed, it seems likely that the stabilization is due to the inhibition of hydroxylation, as we have demonstrated here.

We have found, by GST pulldown assay and immunoprecipitation, that PHDs for HIF1A bind to EBV-encoded proteins involved in growth transformation. Thus, PHD2 binds to EBNA-3 and PHD1 to EBNA-5; this may impair their catalytic activity by steric hindrance. When the cells were subjected to conditions mimicking hypoxia, a small proportion of the available HIF1A was hydroxylated and the levels of both PHD1 and PHD2 increased. Despite binding to EBNA-3, HIF1A was able to transactivate its target genes, including those involved in the glycolytic pathway.

PDK1 phosphorylates the mitochondrial pyruvate dehydrogenase complex responsible for oxidative decarboxylation of pyruvate. The higher level of PDK1 in LCLs compared with mitogen-activated B-cells suggests that the oxidative decarboxylation of pyruvate may be slower in LCLs. The level of GLUT1, a glucose transporter responsible for glucose uptake, is high in thyroid [19] and colorectal cancers [20]. PGK1, a phosphoglycerate kinase, is secreted by fibrosarcoma cells at levels several-fold higher than for normal fibroblasts [21]. PKLR catalyzes the conversion of phosphoenolpyruvate and ADP into pyruvate and ATP in the last step of glycolysis. Hexokinase (HK) phosphorylates glucose into glucose-6-phosphate [22], which is the first step of the glycolytic pathway.

At high oxygen concentration, glucose is metabolized to CO_2_ and the cells produce ATP (about 36 molecules per molecule of glucose) and precursors for amino acid synthesis, whereas only minimal amounts of lactate are generated from pyruvate (reviewed in [23]). At low oxygen concentrations, the cells convert most of the pyruvate to lactate and much less ATP is generated (2–4 molecules per 1 molecule of glucose), in a process referred to as anaerobic glycolysis. However, the rate of ATP production is higher and overall metabolism is more efficient during glycolysis (reviewed in [24]).

It has been shown that tumor cells and rapidly proliferating mitogen-activated T-cells (reviewed in [10], [25]) prefer glycolysis even under normoxic conditions; this phenomenon is usually referred to as the Warburg effect. As a result, the cells can produce more NADH (reduced form of the nicotinamide adenine dinucleotide), intermediates for the synthesis of fatty acids, nonessential amino acids and sugars (for nucleotides); this is believed to facilitate cellular protein synthesis.

Mitogen- (concanavalin A and IL2) activated rat thymocytes metabolize 90% of their glucose to lactate, whereas nonproliferating thymocytes use only 56% of their glucose supply for lactate production [25]. Thus, only 1.1% of available glucose was oxidized through glycolysis to CO_2_ by mitogen-activated cells, whereas there was a 27% conversion in resting cells. Consistent with the high rate of anaerobic glycolysis in rapidly proliferating cells, the activities of hexokinase, 6-phosphofructokinase (PFK) and PKLR were increased 10–30-fold relative to their resting state [25].

Many enzymes involved in glycolysis are the products of HIF1A-responsive genes. These include *GLUT1*, *PFK*, *LDHA1, MCT4 (SLC16A3)* and *PDK1* (reviewed in [26]). Our findings indicate that under normoxic conditions, aerobic glycolysis is active in LCLs and in freshly EBV-infected B-cells, but not in mitogen-activated B-cells.

It has been shown that activation of AKT signaling also results in the induction of aerobic glycolysis [24], [26], [27], and it was proposed that the AKT pathway could be activated by LMP1 (upon CD40 mimicking, discussed in [28]). Recently, it was shown that the AKT pathway is also activated in EBV-transformed B-cells where it counteracts apoptosis and favors cell survival [29].

LCLs produce at least 15 times more ROS, as a by-product of oxidative metabolism, than mitogen-activated B-cells, and EBV-positive Burkitt's lymphoma cells produce higher quantities of ROS than EBV-negative Burkitt's lymphoma cells [30]. LCLs produce much higher levels of ROS than mitogen-activated B-cells without any apparent detrimental effects on the cells. However, LCLs express high levels of LMP1, an EBV-encoded anti-apoptotic protein. EBNA-5 binds to MDM2 and forms trimolecular complexes with p53, thereby inhibiting the p53 transcriptional pathway. This in turn may counteract the DNA-damaging effect of ROS.

Importantly, our findings indicate that EBV-transformed lymphoblastoid cells that have never been exposed to hypoxia exhibit the Warburg effect, a phenomenon that is characteristic of tumor cells.

### Conclusion and hypothesis

**Figure 7:**
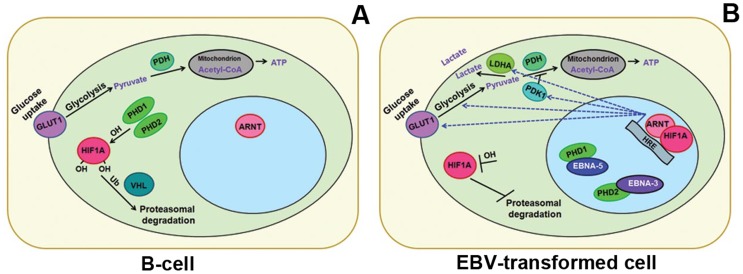
Schematic view on the role of HIF1A in EBV-infected B-cell. **A** – HIF1A is hydroxylated by the PHDs under normoxic conditions. The hydroxylated HIF1A is recognized by the VHL, E3 ubiquitin ligase, and HIF1A is degraded on proteasomes in activated B-cells. **B –** Upon EBV infection, EBNA-3 and EBNA-5 bind to PHD2 and PHD1, respectively, and inhibit HIF1A hydroxylation and degradation. The stabilized HIF1A translocates to the nucleus, forms a heterodimer with ARNT and transactivates genes such as *GLUT1*, *PDK1* and *LDHA*. This results in conversion of pyruvate to lactate, i.e., aerobic glycolysis.

EBV-transformed B-cells stabilize the HIF1A protein, whereas mitogen-activated B-cells do not. The transformed cells use both the aerobic and nonaerobic glycolytic pathways for energy production, even in the presence of abundant oxygen (Figure 7). The virally transformed lymphoblastoid cells thus resemble most solid tumors with regard to the “Warburg effect”.
